# Adsorption Capacities of Iron Hydroxide for Arsenate and Arsenite Removal from Water by Chemical Coagulation: Kinetics, Thermodynamics and Equilibrium Studies

**DOI:** 10.3390/molecules26227046

**Published:** 2021-11-22

**Authors:** Muhammad Ali Inam, Rizwan Khan, Kang Hoon Lee, Muhammad Akram, Zameer Ahmed, Ki Gang Lee, Young Min Wie

**Affiliations:** 1 Institute of Environmental Sciences and Engineering (IESE), School of Civil and Environmental Engineering (SCEE), National University of Sciences and Technology (NUST) H-12 Campus, Islamabad 44000, Pakistan; ainam@iese.nust.edu.pk; 2 Department of Chemical Engineering, Quaid-e-Awam University of Engineering, Science and Technology (QUEST), Nawabshah 67480, Pakistan; rizwansoomro@quest.edu.pk (R.K.); zameerahmedwassan@gmail.com (Z.A.); 3 Department of Civil and Environmental Engineering, Hanyang University, 222 Seongdong-gu, Seoul 04763, Korea; 4 State Key Laboratory of Applied Organic Chemistry, Laboratory of Special Function Materials and Structure Design of the Ministry of Education, College of Chemistry and Chemical Engineering, Lanzhou University, Lanzhou 730000, China; akram@lzu.edu.cn; 5 Department of Materials Engineering, Kyonggi University, Suwon 16227, Korea; gglee@kyonggi.ac.kr (K.G.L.); supreme98@kyonggi.ac.kr (Y.M.W.)

**Keywords:** arsenic sorption, coagulation, environmental behavior, interfering species, iron hydroxide, mechanisms

## Abstract

Arsenic (As)-laden wastewater may pose a threat to biodiversity when released into soil and water bodies without treatment. The current study investigated the sorption properties of both As(III, V) oxyanions onto iron hydroxide (FHO) by chemical coagulation. The potential mechanisms were identified using the adsorption models, ζ-potential, X-ray diffraction (XRD) and Fourier Transform Infrared Spectrometry (FT-IR) analysis. The results indicate that the sorption kinetics of pentavalent and trivalent As species closely followed the pseudo-second-order model, and the adsorption rates of both toxicants were remarkably governed by pH as well as the quantity of FHO in suspension. Notably, the FHO formation was directly related to the amount of ferric chloride (FC) coagulant added in the solution. The sorption isotherm results show a better maximum sorption capacity for pentavalent As ions than trivalent species, with the same amount of FHO in the suspensions. The thermodynamic study suggests that the sorption process was spontaneously exothermic with increased randomness. The ζ-potential, FT-IR and XRD analyses confirm that a strong Fe-O bond with As(V) and the closeness of the surface potential of the bonded complex to the point of zero charge (pH_zpc_) resulted in the higher adsorption affinity of pentavalent As species than trivalent ions in most aquatic conditions. Moreover, the presence of sulfates, phosphates, and humic and salicylic acid significantly affected the As(III, V) sorption performance by altering the surface properties of Fe precipitates. The combined effect of charge neutralization, complexation, oxidation and multilayer chemisorption was identified as a major removal mechanism. These findings may provide some understanding regarding the fate, transport and adsorption properties onto FHO of As oxyanions in a complex water environment.

## 1. Introduction

The emission of arsenic (As)-contaminated wastewater from industries, mining sites and smelting areas into fresh water bodies has raised serious concerns owing to its harmful effects on human health and its destruction of the surrounding environment [1,2]. The environmental behavior of As oxyanions strongly depends upon system redox and pH. Among the four oxidation states of As, the pentavalent form (As(V)) predominates in aerobic environments, whereas the trivalent form (As(III)) occurs in reduced conditions, yet their chemistry, toxicity and sorption affinity are markedly different [3]. For instance, As(III) species exists as H_3_AsO_3_ (pH < 9.22) and H_2_AsO_3_^−^ (9.22 < pH < 13.4), while As(V) ions occur as H_3_AsO_4_ (pH < 2.2), H_2_AsO_4_^−^ (2.2 < pH < 6.97) and HAsO_4_^−2^ (6.97 < pH < 11.53) in aqueous environment [2]. In addition, As(III) is ten times more toxic and has a weaker sorption affinity to sorbents than As(V) ions [2]. Therefore, various environmental agencies, including the Pakistan Environmental Protection Agency (Pak-EPA), the United States Environmental Protection Agency (USEPA) and the World Health Organization (WHO), have set the regulation limit for As at 10 μg/L [4]. As such, it would be critical, both scientifically and technologically, to develop highly efficient methods for removing both As species from water. 

It is widely known that chemical coagulants and adsorbents are promising for the removal of toxicants from aqueous media. Owing to their relatively low cost compared to equally effective removal methods (such as membrane separation, electrocoagulation etc.), iron-based coagulants are extensively used in potable water facilities for the elimination of toxic elements, including As, from water [2,4,5]. It is obvious that iron precipitation is the primary route for iron hydroxide (FHO) formation during the coagulation process; however, precipitation may be reduced dramatically by non-reductive or reductive routes in a multicomponent environment [6,7]. The reductive pathways mainly comprise protons, organic ligands and inorganic reductants [6,8]. Among them, inorganic reductants such as HAsO_4_^2−^ may have a significant impact on FHO formation by altering the physicochemical environments, i.e., pH, surface complexation reactions, phase transformation, deviation in point of zero charge (pH_zpc_), and Fe^2+^ concentration [9]. Our previous studies [10,11] comprehensively investigated the influence of inorganic ligands such as HAsO_4_^2−^ and Sb(OH)_6_^−^ on FHO formation, with results indicating the dissolution of FHO precipitates in the presence of a greater concentration of pentavalent As and antimony (Sb) contaminants [10]. A more pronounced effect on FHO dissolution was observed under alkaline pH conditions in suspensions containing As(V) species [10]. Despite the evident potential influence of As(V) species on FHO formation in water, the sorption performance of this important system has rarely been explored by environmental scholars. Therefore, it is imperative to understand the geochemical, environmental and adsorption behavior onto FHO of As oxyanions in various water samples.

In recent years, the differences in chemistry and environmental behavior of trivalent and pentavalent As species have received substantial attention [10,12,13,14,15]. Iron minerals such as FHO play a crucial role in affecting the transformation and migration of As oxyanions in aqueous media, owing to their poorly crystalline structure with highly reactive vacancy sites, large surface areas and strong adsorption properties [10,16,17]. Several studies have indicated the key role of environmental and chemical conditions, such as contaminant redox form, pH, organic matter and ionic strength, etc., in As mobility in water [10,15,18]. For instance, in our previous study [10], a significant decline in As(V) removal was observed under basic environmental conditions owing to the dissolution of Fe precipitates, while a strong adsorption potential of FHO for As(V) was observed at pH 5–7. Another study indicated that the presence of divalent cations enhances the coagulation performance of As species in water [18]. A remarkable reduction in As removal was observed in the presence of hydrophobic organic ligands during the coagulation process [15,19]. However, how the sorption properties of FHO for As(III, V) species vary with temperature needs significant attention. Several studies have also focused on the sorption potential and removal mechanism of As oxyanions onto and from FHO by coagulation [10,20]. Still, the literature comparing the sorption capacity and rate of FHO for trivalent and pentavalent As oxyanions in complex waters seems scarce. Therefore, understanding the potential mechanisms of As species sorption onto FHO under environmentally relevant conditions is worthy of consideration.

The main aim of the present study was to explore the sorption behavior of As oxyanions onto FHO via chemical coagulation process. In addition, the As sorption kinetics, thermodynamics and isothermal properties were also discussed in detail. The influence of pH and FC dosage on reaction kinetics was also studied. Finally, ζ-potential, X-ray diffraction (XRD) spectrum and Fourier transform infrared (FT-IR) spectrometry were used to explicate the removal mechanism of both contaminants by FHO from water.

## 2. Results and Discussion

### 2.1. Influence of pH on As(III, V) Sorption

**Figure 1:**
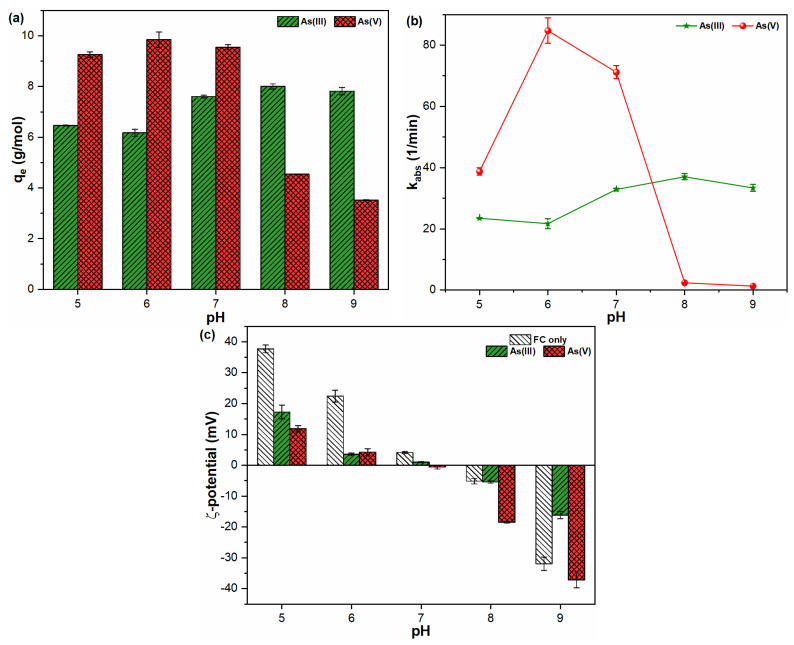
The influence of pH on (**a**) adsorption capacity; (**b**) reaction rate; and (**c**) ζ-potential of FHO with As(III, V) oxyanions.

The solution pH is one of the most critical influential factors that affect the dissolution behavior and transport of metal ions in water. Figure 1 indicates the influence of pH on the sorption capacity, rate and ζ-potential of FHO with As(III, V) species. As presented in Figure 1a, the sorption of trivalent As on FHO showed an increasing trend upon increasing solution pH. Such results are in good agreement with the fact that the first dissociation constant of the H_3_AsO_3_ species is 9.22, and As(III) acts as a Lewis base, hence it can interact with Lewis acid, i.e., FHO precipitates across a wide pH range [21,22]. Moreover, the As(III) adsorption results were well supported by the high adsorption rate of As(III) ions with pH and ζ-potential values close to pH_zpc_ under similar aquatic conditions (Figure 1b,c). In contrast, the As(V) species showed good adsorption capacities in the pH range of 5–7, with poor adsorption performance above pH 7 (Figure 1a). This might be related to the sufficient FHO precipitation in acidic to neutral pH conditions (Figure S1), as a result of electrostatic attraction between positively charged Fe(III) complexes and negatively charged As(V) ions [23,24,25]. A higher k_abs_ for As(V) than As(III) was observed in experimental pH ranges (5–7), and the sorption rate of pentavalent As was 1.5–4.0 times faster than trivalent As under similar experimental conditions (Figure 1b). The ζ-potential values of FHO-As(III, V) came closer to pH_zpc_ in similar aqueous media, when compared with suspensions containing FHO precipitates only (denoted as FC only (Figure 1c)). However, the alkaline pH conditions were found to be unfavorable for the elimination of pentavalent As species from aqueous matrices. This might be attributed to the dissolution of FHO precipitates, the slowdown of the adsorption process and the decline in ζ-potential values towards a more negative trajectory (Figure S1 and Figure 1b,c). Moreover, the higher binding energy of the As(V) system in alkaline environments may support our finding that complexation between anionic As(V) and FHO species becomes difficult, resulting in higher residual As(V) ions in water [26]. In general, these findings suggest that the suspension’s pH might have a significant impact on the surface characteristics and stability of FHO precipitates in suspensions containing As(III, V) species, thus affecting the adsorption rates of both toxicants on the FHO surface. Similarly, previous studies [12,18,27] have also indicated that solution chemistry is mainly responsible for changing the physicochemical properties of early formed FHO precipitates, thereby affecting the sorption properties of As oxyanions in water.

### 2.2. Adsorption Kinetics and Reaction Rate

**Figure 2:**
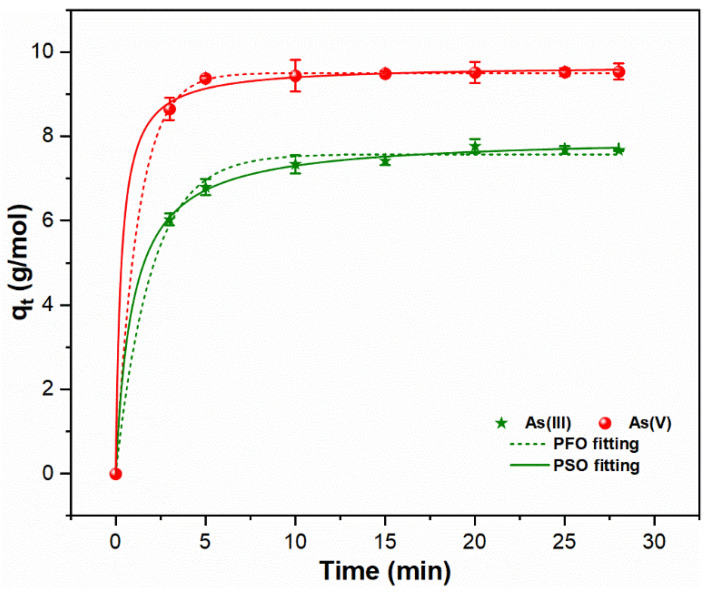
Adsorption kinetics data and corresponding PFO and PSO fitting for the removal of As(III, V) oxyanions during ferric chloride coagulation.

The reaction rate is the crucial factor that affects the mobility and control of As(III, V) species in aqueous media. Figure 2 highlights the variation in kinetic curves for both As oxyanions on the FHO surface during chemical coagulation. A higher adsorption capacity q_t_ of As(V) (8.65 g/mol) than As(III) (6.03 g/mol) was observed during the sweep coagulation phase, i.e., 3 min. This might be due to the electrostatic attractive forces between HAsO_4_^2−^ and Fe(OH)_2_^+^ species [18,22]. It was also notable that during sweep coagulation, more FHO precipitation was observed in the As(V) system; however, a similar quantity of FHO precipitates was formed in both As(III, V) suspensions during the flocculation process (Figure S2). Equilibrium sorption for trivalent and pentavalent As was achieved within 20 min of the flocculation time, with q_e_ of 7.996 and 9.685 g/mol, respectively (Table 1). These findings suggest that the sorption rate and capacity of pentavalent As on FHO are better than the trivalent species. Similarly, a previous study [10] also indicated the higher adsorption ability of pentavalent As species than trivalent As ions during the chemical coagulation process.

Additionally, the applicability of PFO and PSO models were examined using kinetic data to explore the dominant mechanism involved in the adsorption of As species onto precipitated FHO (Figure 2). The experimental and model parameters are presented in Table 1. It is obvious that the regression coefficient (R^2^) of PSO (As(III, V):0.999) is slightly greater than that of PFO (As(III):0.996, As(V):0.999)), and the experimental q_e_ values for both As oxyanions are much closer to the calculated PSO q_e_ values. A smaller k_2_ value was observed for As(III) species (0.132 mol/g.min) than for As(V) ions (0.342 mol/g.min). Therefore, the adsorption properties of both As oxyanions can be effectively explained by the PSO model, which suggests the possibility of both physisorption and chemisorption reactions between the FHO and As species in water [28,29].

### 2.3. Influence of FC Dosage on As(III, V) Sorption

**Figure 3:**
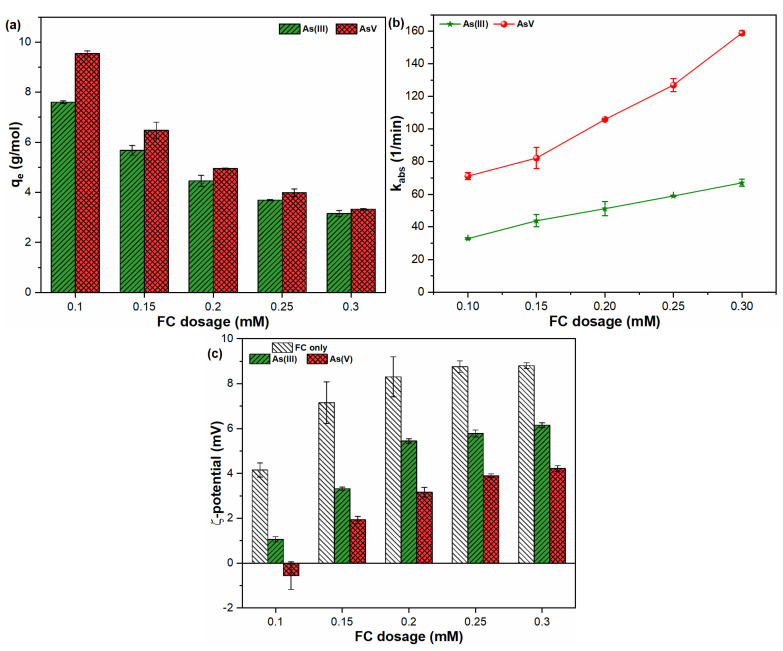
The influence of FC dosages on (**a**) adsorption capacity; (**b**) reaction rate; and (**c**) ζ-potential of FHO with As(III, V) oxyanions.

The coagulant dose has been recognized as a significant factor for determining the As removal efficiency during potable water treatment operations. Figure 3 indicates the influence of FC dosage on the sorption capacity, rate, and ζ-potential of FHO with As(III, V) species. As shown in Figure 3a, higher sorption affinities were observed for the pentavalent As species than the trivalent one, and this decreased with increasing FC doses for both contaminants. Such behavior may be related to the higher level of FHO precipitate formation with increasing coagulant dose (Figure S3), thereby providing more FHO surface sites to the As species [30]. Similar results were derived in earlier studies [31,32,33]. The reaction rates for both As(III, V) species with FC were also examined (Figure 3b). The kabs for As(III, V) oxyanions indicated an increasing trend with increasing FC dosages, indicating that the kinetics of both toxicants are highly dependent on FHO precipitates. Similarly, a positive dependency of FC concentration on heavy metals adsorption was reported in our earlier study [10]. It is noteworthy that the k_abs_ of the anionic As(V) species was significantly greater than that observed for neutral As(III) ions, and that much higher k_abs_ values were observed at a higher dosage gradient of FC (Figure 3b). The k_abs_ of As(III) increased from 32.87 (1/min) to 67.05 (1/min) when the applied FC dosages were increased from 0.10 mM to 0.30 mM. Further, the k_abs_ of As(V) was 1.88–2.37 times higher than that of the As(III) species. Therefore, it can be inferred that the sorption rate of pentavalent As onto FHO was faster than that of trivalent As ions. 

The surface charge of FHO precipitates was examined in pure water and As(III, V) suspensions (Figure 3c). It was observed that the presence of As oxyanions brings the surface potential of FHO closer to pH_zpc_. These results suggest that the electrostatic attractive forces of FHO increase in solutions containing As(III, V) species when compared with pure water. Therefore, higher adsorption rates were observed at higher FC dosages (Figure 3b). In addition, As(V) suspensions presented a greater lowering of ζ-potential for FHO than As(III) solutions (Figure 3c). These results might be attributable to the strong interaction of anionic As(V) ions with cationic FHO precipitates, when compared with neutral As(III) ions [18,34]. The difference in adsorption properties of both contaminants may also be related to the lower binding energy of pentavalent As than trivalent ions at the solid–liquid interface, thus decreasing the ζ-potential and enhancing the adsorption strength as well as the rate of As(V) in water [12]. In general, these results indicate that increasing the coagulant dosage may improve particle agglomeration kinetics and increase pollutant adsorption rates during water treatment operations.

### 2.4. Adsorption Isotherm 

**Figure 4:**
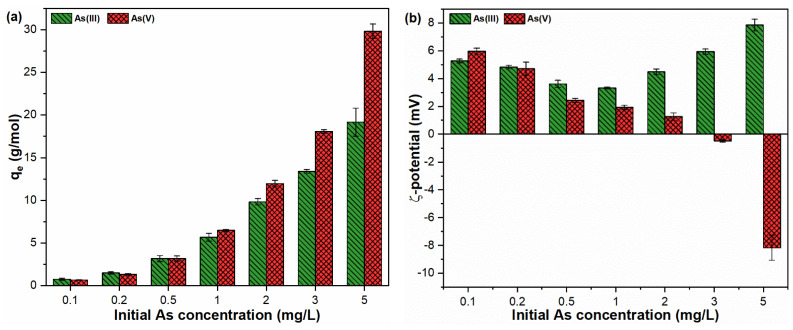

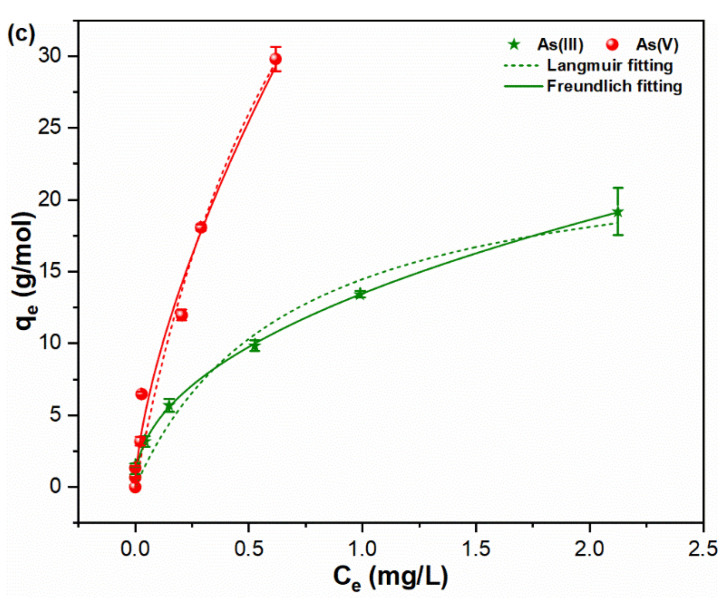
(**a**) Adsorption capacity; (**b**) ζ-potential; (**c**) Langmuir and Freundlich sorption isotherms for As(III, V) by FHO.

Sorption isotherm models are frequently applied to understand the distribution of adsorbed molecules between the solid and liquid phases under equilibrium conditions. The results of sorption experiments employing various initial As(III, V) concentrations at neutral pH are presented in Figure 4a. The sorption abilities for both trivalent and pentavalent As oxyanions increased remarkably upon increasing the initial toxicants loading from 0.1 to 5 mg/L (Figure 4a). The As(III, V) suspensions showed similar quantities of FHO formation under the studied conditions (Figure S4). The surface potentials of FHO in As(III, V) suspensions were also examined, with the results indicating a continuous decrease in ζ-potential values upon increasing As(V) concentration (Figure 4b). The As(V) ions predominantly exist as HAsO_4_^2−^ in a neutral pH environment [27,35]. Therefore, a shift in ζ-potential values from positive to negative was seen in suspensions containing higher As(V) loading (Figure 4b). The sorption data fitted with Langmuir and Freundlich isotherm models (Figure 4c). The experimental and model fitting parameters are presented in Table 2. For the sorption of As oxyanions onto FHO, the Freundlich model better describes the adsorption process, as determined by the R^2^ values (As(III):0.999, As(V):0.902). The k_F_ value observed for the As(V) species was three times higher than As(III), indicating the strong sorption affinity of As(V) species towards FHO precipitates. Moreover, a lower n value, i.e., 1.744 for As(V), was observed than the 2.168 for As(III), showing the faster adsorption of As(V) than As(III) onto FHO in water. Such observations are consistent with our previous findings that the adsorption properties of FHO for As(V) perform well when compared with As(III) species. Overall, these results suggest that FHO showed a heterogeneous surface of adsorption sites with multilayer adsorption characteristics for both As oxyanions in water [36,37].

In order to compare the maximum adsorption capacity of FHO for the As(III, V) oxyanions, Table 3 indicates the other reported sorption affinities of iron-based low-cost adsorbents for both toxicants. It can be clearly seen that the FHO precipitates formed in the current study presented much better maximum adsorption capacities than any other sorbent reported to date, which further favors the coagulation-induced adsorption process for arsenic remediation and general water treatment applications.

### 2.5. Adsorption Thermodynamics

**Figure 5:**
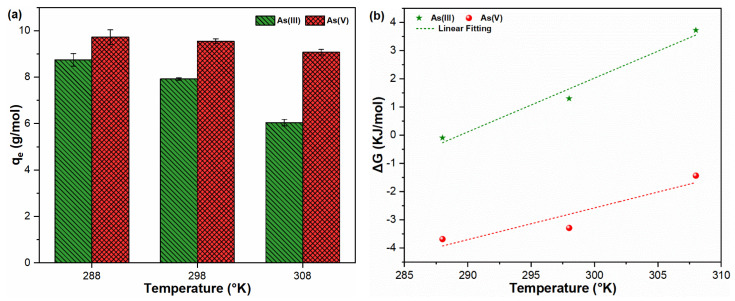
(**a**) Adsorption capacity and (**b**) Van’t Hoff plot for sorption of As(III, V) oxyanions onto FHO at varying temperatures.

Variations in solution temperature may affect the sorption reactions of contaminants with adsorbents in an aqueous media. Figure 5a indicates the effect of temperature on the sorption properties of As oxyanions onto FHO. The remarkable decrease in As(III) sorption capacity at higher temperatures is probably due to the lower level of FHO precipitation under such conditions (Figure 5a and Figure S5). This might be related to the weakening and breaking of the Fe-O and As(III)-Fe bonds as a result of the increased thermal energy at higher temperatures [42,43]. In contrast, no substantial effect on FHO precipitation and As(V) adsorption was observed in the studied temperature range (Figure 5a and Figure S5).

The thermodynamic parameters were calculated to further clarify the sorption characteristics of As oxyanions in a water environment (Figure 5b and Table 4). The ∆G° at given temperatures with negative values indicated spontaneous characteristics and the feasibility of adsorption reactions between As(III, V) and FHO [44]. The negative values of ∆H° illustrated that the adsorption of As(III, V) onto FHO is favorable owing to the exothermic characteristics of the adsorption process [45]. It has been previously discussed that the values of adsorption energy <8 KJ/mol, 8–16 KJ/mol and >16 KJ/mol indicate physisorption, ion exchange and chemisorption phenomena, respectively [46,47]. Absolute ∆H° values of 55.188 (As(III)) and 36.31 (As(V)) were observed, indicating chemisorption as the major binding mechanism between FHO and both toxicants in water. Moreover, the positive values of ∆S° showed that randomness increases upon increasing temperature during the adsorption process [43]. These results could be helpful in understanding the adsorption reactions between FHO and As species during the chemical coagulation process. 

### 2.6. Influence of Interfering Ions on As(III, V) Sorption

**Figure 6:**
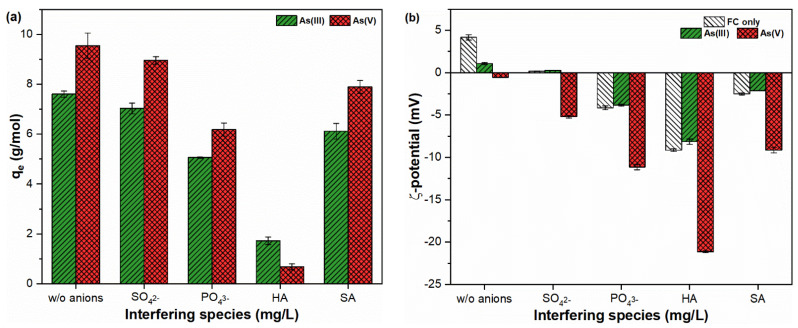
(**a**) Adsorption capacity and (**b**) ζ-potential of FHO in suspensions containing As(III, V) ions and interfering species.

Figure 6 indicates the sorption affinity and ζ-potential for FHO of As species under the influence of various anionic species, including sulfate, phosphate, and humic and salicylic acid. It was observed that the presence of anionic species resulted in decreases in the sorption capacity of As oxyanions (Figure 6a). For instance, sulfates compete for the sorption sites of FHO with both As(III, V) species; however, their impacts on As sorption were observed to be the lowest as compared to other competing ions. Similarly, phosphate had a competitive inhibitory effect on As sorption, which is consistent with earlier studies [48,49]. As shown in Figure 6a, the presence of humic acid remarkably decreases the level of As sorption onto FHO surfaces. Such inhibitory behavior may be due to the higher sorption ability of HA molecules for FHO than As oxyanions [50]. In addition, it was noted that the HA molecules significantly direct the ζ-potential values towards a more negative trajectory (Figure 6b). This in turn increases the electron density between Fe and As molecules, resulting in the dissolution of FHO in suspensions co-contaminated with As species and HA molecules (Figure S6). Hydrophilic SA molecules, on the other hand, had a smaller impact on As(III, V) sorption onto the FHO surface (Figure 6a). The presence of low-molecular weight compounds and weaker acidic groups in SA might result in less interactive behavior with FHO surface sites [51]. Overall, the sorption ability of As oxyanions onto FHO can be ranked in the following order: without anions > SO_4_^2−^ > SA > PO_4_^3−^ > HA.

### 2.7. Mechanism of As(III, V) Adsorption onto FHO

**Figure 7:**
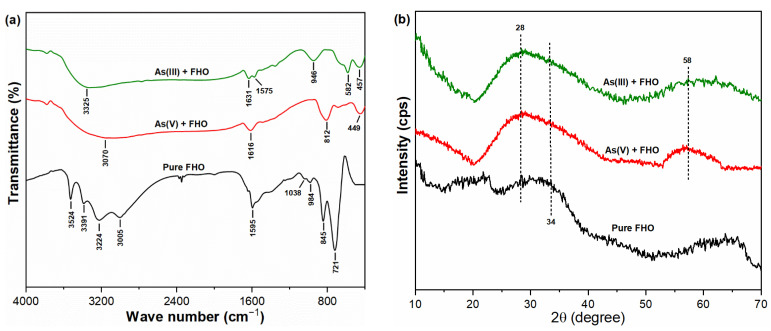
(**a**) FT-IR spectra and (**b**) XRD patterns of FHO before and after reaction with As(III, V) oxyanions.

The surface states of FHO before and after the sorption of both As oxyanions were studied using different spectrum techniques, including FT-IR and XRD, to explore the sorption mechanism of both contaminants on the FHO surface (Figure 7). The IR spectra of FHO before and after the adsorption of As(III, V) oxyanions is presented in Figure 7a. The main peak observed for pure FHO at ~721 cm^−1^ was subjected to Fe-O bending vibrations [52]. The band observed in pure FHO in the range 3000–3550 cm^−1^ might be ascribed to the stretching vibrations of OH [37]. In comparison, the intense peaks observed at ~3070 and ~3325 cm^−1^ were ascribed to the polar interactions of FHO-hydrolyzed products with As(III, V) species in water. The shift in the peaks from ~1595 cm^−1^ to (As(V)) ~1616 cm^−1^ and (As(III)) ~1631 and ~1575 cm^−1^ suggest that As(III, V) species may interact with the FeOH groups and form complexes by deforming water molecules [10]. The peaks appearing at ~812 cm^−1^ and ~582 cm^−1^ may be related to the stretching vibrations of As(V)-O and As(III)-O bonds on the FHO surface, respectively [53,54]. The FT-IR analysis results suggest that the sorption of As(III, V) ions onto FHO may be proceeded by complexation reactions between both As species and FHO precipitates.

To further illustrate the mechanisms employed by FHO for the sorption of As oxyanions, the XRD spectra of FHO before and after reaction with As species were obtained as shown in Figure 7b. The diffraction peak observed at 2θ = 34° showed the interaction of As(III, V) species with FHO precipitates via internal and surface adsorption mechanisms [43]. The fridge pattern centered at 2θ values of 28° and 58° indicates the bonding structures of poorly crystalline ferric arsenate [53,55]. It is worth mentioning that poorly crystalline ferric arsenate was formed after reaction with both As(III, V) oxyanions, which was not evidenced in the FT-IR analysis. The results of XRD analysis for the Fe(III)–As(III)–H_2_O system may be supported by the involvement of the transformation of As(III) to As(V), followed by sorption onto the FHO surface, resulting in the formation of amorphous ferric arsenate [56,57]. For the case of the Fe(III)–As(V)–H_2_O system, the ferric arsenate was formed from FeHAsO_4_^+^ and FeH_2_AsO_4_^2+^ precursors [53]. Thus, the XRD analysis further elucidated the role of the oxidation of As(III) to As(V) following sorption onto the FHO surface. 

In general, the experimental results and analytical investigations suggest that the major binding mechanisms might be the combined effects of the charge neutralization, complexation, oxidation and multilayer chemisorption of As(III, V) oxyanions on the FHO surface during the chemical coagulation process. Similarly, prior research [13,21,27] described comparable mechanisms when working on the conventional treatment of heavy metal ions in water.

### 2.8. Implications for Mobility and Remediation 

Understanding the sorption of As(III, V) oxyanions by iron-based sorbents such as FHO is significant to evaluate their fate, mobility and toxicity in drinking water. Our current study suggests the stronger adsorption potential of As(V) than As(III) in an iron-rich environment, specifically under neutral pH conditions. However, the mobility of As(V) species was greatly enhanced under alkaline pH conditions, as a result of the strong repulsion between oppositely charged FHO and As(V) species. As such, the environmental conditions play a crucial role in identifying the redox form of As oxyanions in contaminated areas. Despite the variety of sorption properties of As oxyanions, FHO still showed a better ability to remove these pollutants from contaminated media via various sorption mechanisms. Considering chemical coagulation as a cost-effective and efficient technique, an appropriate removal strategy in terms of optimum coagulant dosage should be adopted for As oxyanion removal from complex water environments. 

## 3. Materials and Methods

### 3.1. Materials

The reagent-grade chemicals including arsenic trioxide (As_2_O_3_), humic acid (HA), salicylic acid (SA) and sodium arsenate dibasic heptahydrate (Na_2_HAsO_4_.7H_2_O) were purchased from Sigma Aldrich (St. Louis, MO, USA). The other chemicals, including nitric acid (HNO_3_), sodium hydroxide (NaOH), hydrochloric acid (HCl), sodium dihydrogen phosphate (NaH_2_PO_4_.H_2_O), magnesium sulfate (MgSO_4_.7H_2_O) and ferric chloride hexahydrate (FeCl_3_.6H_2_O), were procured from local suppliers. The pure water from Millipore water purification system (Milli-Q, Millipore Co., Bedford, MA, USA) was used to prepare stock solutions and synthetic test samples. Before use, all glassware was washed with 15% HNO_3_ followed by rinsing with pure water to avoid contamination. The stock solutions of As(III) and As(V) at 100 mg/L were prepared separately by dissolving As_2_O_3_ and Na_2_HAsO_4_.7H_2_O in 1 M NaOH solution and pure water, respectively. The stock solutions of hydrophobic HA and hydrophilic SA were prepared by dissolving 500 mg chemical in 100 mL pure water. The detailed procedure for HA and SA solution preparation can be found elsewhere [58]. The stock solution of coagulant (100 mM) was prepared using FeCl_3_.6H_2_O. The working solutions of trivalent and pentavalent As were prepared via sequential dilution from each stock solution for each experimental run.

### 3.2. Experimental Design

The jar tester with multiple blades (Young Tech, S., Gyeongsangbuk-Do, Korea) was used to conduct coagulation experiments. The differences in As(III, V) sorption and FHO formation were examined under a series of coagulation experiments. Initially, chemical coagulation experiments were conducted using a 0.1 mM FC dose in 1 mg/L As(III, V) suspensions at various pH (5–9) conditions. The kinetic experiments were also performed for (1 mg/L) As(III, V) suspensions at contact time 0–28 min, pH 7, temperature 298 K and FC dose 0.1 mM. The aliquots were taken after different time intervals (0, 3, 5, 10, 15, 20, 25 and 28 min). The influence of applied FC dosages (0.1–0.3 mM) was also investigated. The sorption isotherm experiments were also conducted at varying As(III, V) concentrations (0–5 mg/L), FC dosages (0.15 mM), pH levels (7), temperatures (298 K) and equilibrium times. In order to examine the influence of temperature on the sorption process, a thermodynamic study was performed at different temperatures (288 K, 298 K and 308 K) with FC dosage 0.1 mM, As(III, V) concentration 1 mg/L and pH 7. The pH was set using 100 mM HCl and NaOH for all experiments, while temperature was monitored using a thermometer. The influence of various interfering species, i.e., sulfates (50 mg/L), phosphates (0.5 mg/L), and humic and salicylic acid (10 mg/L each), was also examined. The residual As(III, V) and Fe(III) concentrations were analyzed after filtering the supernatant using a 0.45µm glass filter. For all chemical coagulation experiments, the sequential experimental strategy was as follows: 3 min fast agitation (coagulation); 20 min slow mixing; 30 min quiescent settling; and aliquot collection [20]. However, only slow mixing for 25 min was done for kinetic experiments, with the rest of the experimental protocol remaining same. All experiments were performed in triplicates and data were shown with error bars showing standard deviations.

### 3.3. Modeling Coagulation Data by Sorption Studies

The amount of FHO formed (C_s_ (mol/L)) by FC coagulation of As(III, V) suspension was calculated using Equation (1):

(1) $$\mathrm{FHO}:C_{s}={\;I}_{o}-I_{e}$$

where I_o_ and I_e_ (mol/L) represents initial and residual Fe(III) concentration, respectively, in suspensions. The adsorption capacity (q_e_ (g/mol)) of FHO for As(III, V) species is expressed as Equation (2):

(2) $$\mathrm{Adsorption}\;\mathrm{capacity}:{\;q}_{e}=\frac{C_{o}-C_{e}}{C_{s}}$$

where C_e_ and C_o_ (mg/L) indicate the residual and initial As(III, V) concentration, respectively. In order to extend the understanding of As sorption onto FHO, sorption kinetics data were fitted with non-linear pseudo-first order (PFO) (Equation (3)) and pseudo-second-order (PSO) (Equation (4)) models [23,59], as shown below:

(3) $$\mathrm{PFO}:{\;q}_{t}={\;q}_{e}-e^{{\mathrm{lnq}}_{e}-{\mathrm{tk}}_{1}}$$

(4) $$\mathrm{PSO}:{\;q}_{t}=\frac{k_{2}q_{e}^{2}t}{1+k_{2}q_{e}t}$$

where q_e_ and q_t_ (g/mol) represent adsorption capacity of As oxyanions at equilibrium and various time intervals, respectively; t (min) indicates contact time; and k_1_ (1/min) and k_2_ (mol/g.min) indicate rate constants of PFO and PSO models, respectively. In order to investigate the reaction kinetics of As species with the application of pH and FC dosages, Equation (5) was used to explain the sorption process of As species on the FHO surface [12].

(5) $$\mathrm{Rate}\;\mathrm{Constant}:\;\ln\left(\frac{C_{t}}{C_{o}}\right)=k_{\mathrm{abs}}t$$

where k_abs_ (1/min) indicates the rate constant under applied FC dosage and adjusted pH conditions. To further derive the mechanistic insights into the general sorption process, the two most commonly used sorption isotherms were applied to experimental data using Langmuir (Equation (6)) and Freundlich (Equation (7)) models [2,60]:

(6) $$\mathrm{Langmuir}:\;q_{e}=\frac{q_{\max}k_{L}C_{e}}{1+k_{L}C_{e}}$$

(7) $$\mathrm{Freundlich}:\;q_{e}=k_{F}C_{e}^{\frac{1}{n}}$$

where q_m_ (g/mol) refers to the maximum As(III, V) adsorption capacity, n is the index for heterogeneity of the FHO surface, and k_L_ (L/mg) and k_F_ [(g/mol)(L/mg)]^1/n^ indicate Langmuir and Freundlich adsorption affinity constants, respectively. Since solution temperature plays a vital role in the sorption and bonding mechanism of pollutants towards adsorbents, the thermodynamic parameters were calculated at temperatures of 15–35 °C using the Van’t Hoff method as presented in Equations (8–9) [59,61].

(8) $$\mathrm{Distribution}\;\mathrm{Coefficient}:\;k_{d}=\left(\frac{C_{o}-C_{e}}{C_{e}}\right)\times \frac{1}{C_{d}}$$

(9) $$\mathrm{Gibbs}\;\mathrm{free}\;\mathrm{energy}:\;\Delta G=-{\mathrm{RTln}\;k}_{d}$$

(10) Thermodynamic parameters: ΔG=ΔH− TΔS

where k_d_ (L/mg) is the distribution coefficient in As sorption, C_d_ (mg/L as Fe) is the concentration by mass of precipitated FHO, R (8.314 J/mol.K) is universal gas constant and T is reaction temperature (K). Thermodynamic parameters, i.e., ΔG (KJ/mol), ΔH (KJ/mol) and ΔS (KJ/mol.K), represent the Gibbs free energy, enthalpy and entropy of the reaction, respectively. The linear fitting of plot ∆G versus T provides the ΔS (slope) and ΔH (intercept) values using Eq (10).

### 3.4. Analytical Procedures

The residual As(III, V) and Fe(III) concentrations were analyzed using Inductively Coupled Plasma Optical Emission Spectrometry (ICP-OES, Santa Clara, CA, USA). A calibrated pH meter (HACH, Loveland, CO, USA) was used for the pH adjustment of the suspension. The ζ-potential values of the precipitated FHO in suspensions were analyzed using Zetasizer (Malvern, UK). The bonding features of pristine compounds and FHO precipitates after reaction with As(III, V) species were recorded using FT-IR (JASCO Analytical Instruments, Easton, PA, USA) and an X-ray diffractometer (Rigaku, Tokyo, Japan). Moreover, Origin Pro 9.0 (Massachusetts, MA, USA) was used for plotting experimental data points.

## 4. Conclusions

This work demonstrates the kinetics and mechanisms of As(III, V) adsorption onto FHO surfaces by the chemical coagulation process. The experimental results show the stronger sorption ability and faster reaction rate of pentavalent As species in most aquatic conditions, when compared with trivalent ions. At the tested pH values (5–7), the sorption rate of pentavalent As was found to be greater than the trivalent species at the same applied FC dosage; however, the adsorption rate of As(III) was faster than As(V) under alkaline water environments, i.e., pH 9. The FHO precipitation was mainly governed by the quantity of coagulant dosage added, pH, and type of redox As species in solution. The adsorption of both As(III, V) oxyanions onto FHO followed the pseudo-second-order and Freundlich isotherm models. The thermodynamic investigations revealed the spontaneous nature of the reaction between the As species and the FHO surface in most contaminated waters. A change in surface properties of FHO in the presence of interfering species, i.e., sulfates, phosphates, and humic and salicylic acid, was observed, thereby reducing the sorption affinity of As(III, V) ions in such suspensions. Furthermore, the potential mechanisms employed by FHO for As(III, V) adsorption were well described by ζ-potential, FT-IR and XRD analyses, indicating the combined effect of charge neutralization, complexation, oxidation and multilayer chemisorption. These findings suggest that when As species are present in a water environment, the better adsorption affinity for FHO of As(V) would be observed, compared to the As(III) species.

## Figures and Tables

**Table 1:** PFO and PSO kinetic parameters for As(III, V) adsorbed onto FHO during chemical coagulation.

Species	Experimental Parameters			PFO Constants			PSO Constants		
	pH	t (min)	q_e,exp_ (g/mol)	k_1_ (1/min)	q_e,cal_ (g/mol)	R^2^	k_2_ (mol/g.min)	q_e,cal_ (g/mol)	R^2^
As(III)	7	0–28	7.760	0.504	7.575	0.996	0.132	7.996	0.999
As(V)			9.546	0.808	9.505	0.999	0.342	9.685	0.999

**Table 2:** Langmuir and Freundlich isotherm parameters for As(III, V) adsorbed onto FHO during chemical coagulation.

Species	Experimental Parameters		Langmuir Constants			Freundlich Constants		
	pH	FC Dosage (mM)	k_L_ (L/mg)	q_max_ (g/mol)	R^2^	k_F_ ((g/mol)(L/mg))^1/n^	n	R^2^
As(III)	7	0.15	1.487	24.194	0.966	13.472	2.149	0.989
As(V)			1.195	69.558	0.962	40.195	1.518	0.978

**Table 3:** Maximum arsenic (Ⅲ, Ⅴ) sorption capacities (mg/g) reported in the literature on iron-based low-cost adsorbents.

Adsorbents	Concentration Range (mg/L)	pH	Adsorption Capacity (mg/g)		Reference
			As(III)	As(V)	
Fe–sericite composite powder	1–30	5.99–6.11	15.04	13.21	[38]
Fe–sericite composite beads	1–30	6.82–6.85	9.02	7.11	
Iron oxide-impregnated charred granulated attapulgite	0.05–200	7	3.25	5.09	[39]
Waste Fe-Mn oxides embedded in chitosan	10–190	7	44.17	26.80	[40]
Zero-valent iron–biochar complexes (Red oak)	1–25	-	-	15.58	[41]
Zero-valent iron–biochar complexes (Switchgrass)	1–25	-	-	7.92	
Iron hydroxide	0.1–5	7	433.12	1245.45	This study

**Table 4:** Thermodynamic parameters for the sorption of As(III, V) onto FHO during chemical coagulation.

Temperature (K)	∆G (KJ/mol)	∆H (KJ/mol)	∆S (KJ/mol.K)	R^2^
As(III)				
288	−0.093	−55.188	0.191	0.976
298	1.298			
308	3.721			
As(V)				
288	−3.683	−36.31	0.112	0.877
298	−3.288			
308	−1.434			

## Data Availability

All data used to support the findings of this study are included within the article.

## Supplementary Materials

The following are available online, Figure S1: FHO formation across broad pH range in the absence and presence of As(III, V) species, Figure S2: FHO formation as function of contact time, Figure S3: FHO formation under different FC dosages in the absence and presence of As(III, V) oxyanions, Figure S4: FHO formation under different As(III, V) concentrations, Figure S5: FHO formation under varying temperature environment in As(III, V) suspensions, Figure S6: FHO formation under the influence of various interference species.

## Sample Availability

Samples of the compounds are not available from the authors.
