# Supplementary material for: Adsorption Capacities of Iron Hydroxide for Arsenate and Arsenite Removal from Water by Chemical Coagulation: Kinetics, Thermodynamics and Equilibrium Studies
Source: Molecules. 2021 Nov 22;26(22):7046. doi: 10.3390/molecules26227046 (PMC8624347; doi:10.3390/molecules26227046)
Supplement: Supplementary file 1 [file molecules-26-07046-s001.zip › molecules-1455792-supplementary.pdf]

molecules

MDPI

Supplementary Material

Adsorption Capacities of Iron Hydroxide for Arsenate and Arsenite Removal from Water by Chemical Coagulation: Kinetics, Thermodynamics and Equilibrium Studies

Muhammad Ali Inam [1], Rizwan Khan [2], Kang Hoon Lee [3,*], Muhammad Akram [4], Zameer Ahmed [2], Ki Gang Lee [5] and Young Min Wie [5]

[1] Institute of Environmental Sciences and Engineering (IESE), School of Civil and Environmental Engineering (SCEE), National University of Sciences and Technology (NUST) H-12 Campus, Islamabad 44000, Pakistan; ainam@iese.nust.edu.pk
[2] Department of Chemical Engineering, Quaid-e-Awam University of Engineering, Science and Technology (QUEST), Nawabshah, 67480, Pakistan; rizwansoomro@quest.edu.pk (R.K.), zameerahmedwassan@gmail.com (Z.A.)
[3] Department of Civil and Environmental Engineering, Hanyang University, 222 Seongdong-gu, Seoul 04763, Korea
[4] State Key Laboratory of Applied Organic Chemistry, Laboratory of Special Function Materials and Structure Design of the Ministry of Education, College of Chemistry and Chemical Engineering, Lanzhou University, Lanzhou 730000, China; akram@lzu.edu.cn
[5] Department of Materials Engineering, Kyonggi University, Suwon, 16227, Korea; gglee@kyonggi.ac.kr (K.G.L.), supreme98@kyonggi.ac.kr (Y.M.W.)
* Correspondence: diasyong@hanyang.ac.kr

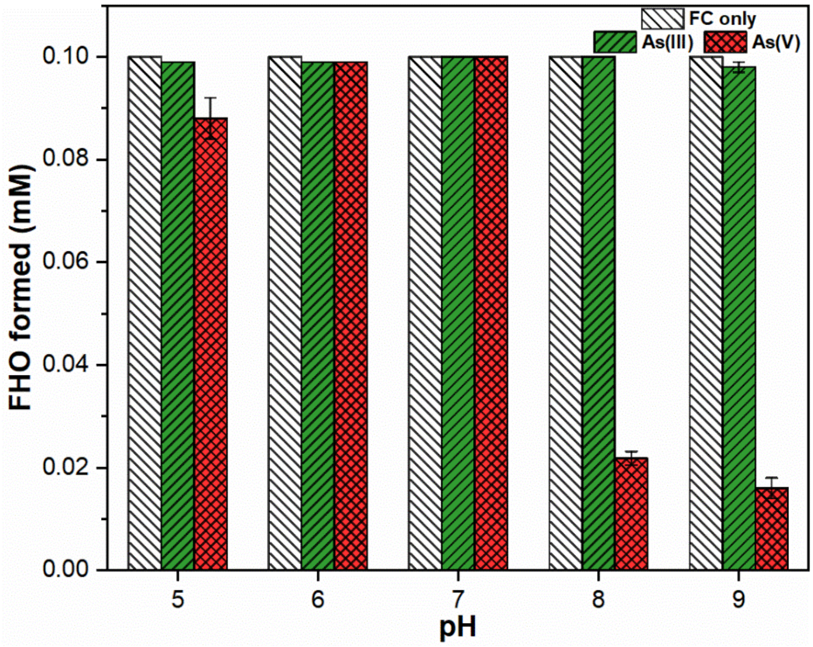

Figure S1. FHO formation across broad pH range in the absence and presence of As(III, V) species.

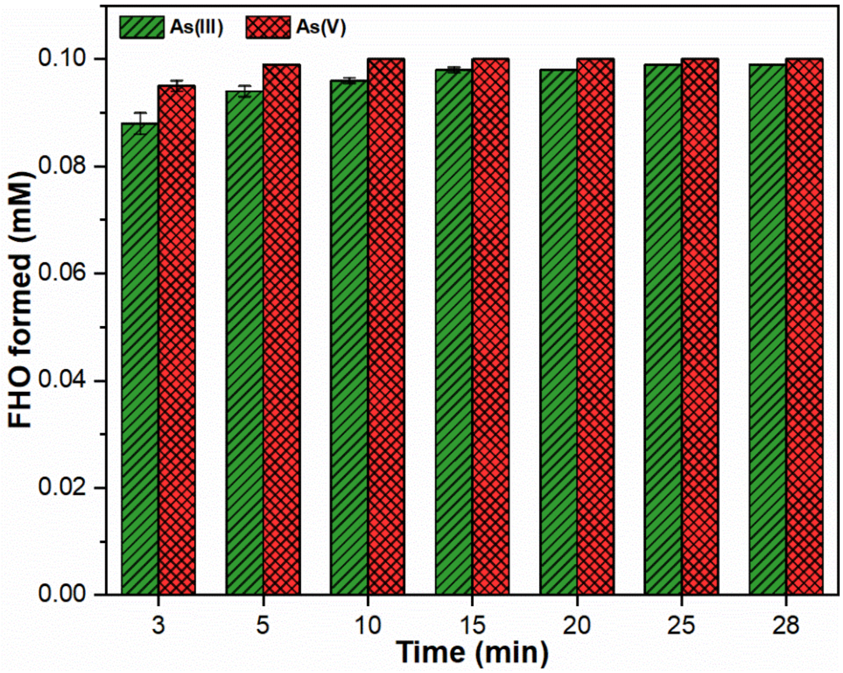

Figure S2. FHO formation as function of contact time.

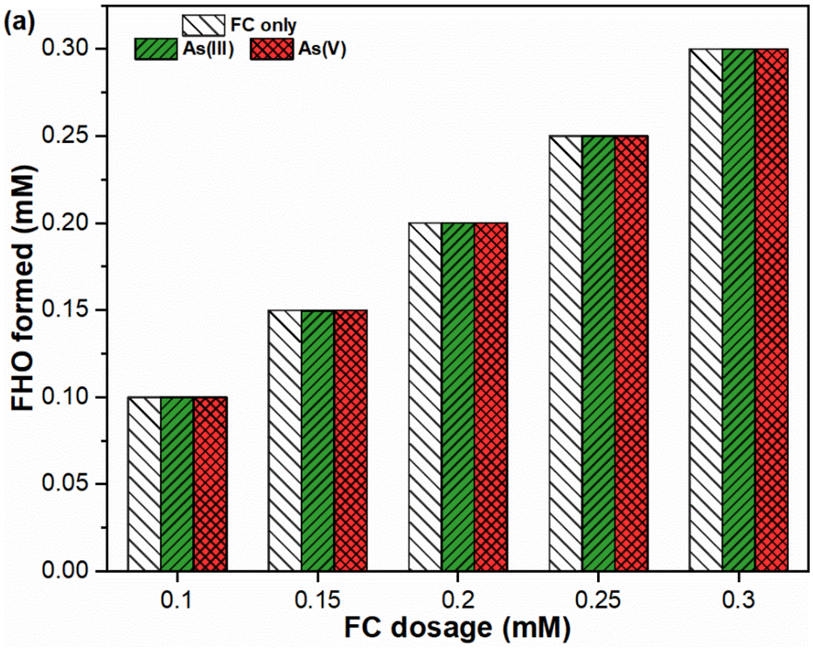

Figure S3. FHO formation under different FC dosages in the absence and presence of As(III, V) oxyanions.

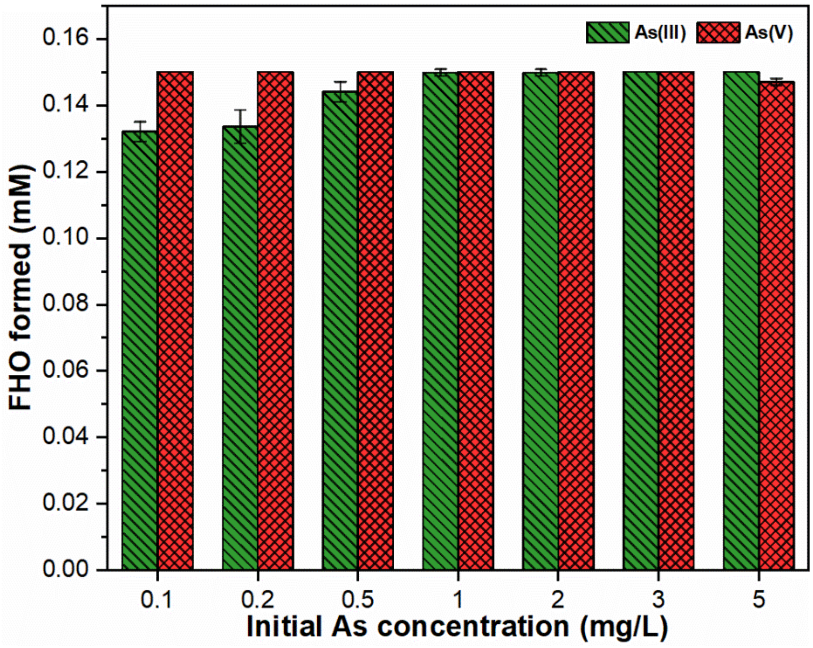

Figure S4. FHO formation under different As(III, V) concentrations.

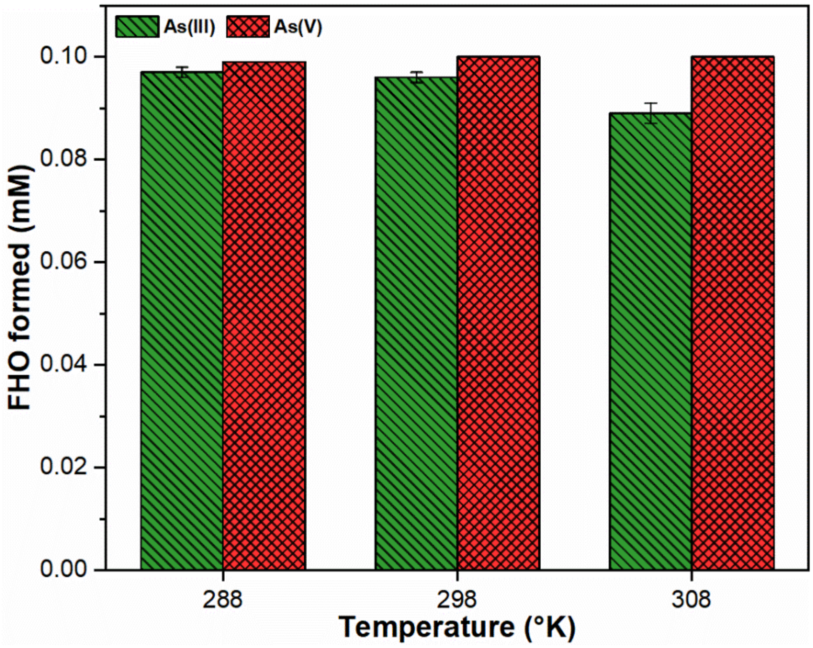

Figure S5. FHO formation under varying temperature environment in As(III, V) suspensions.

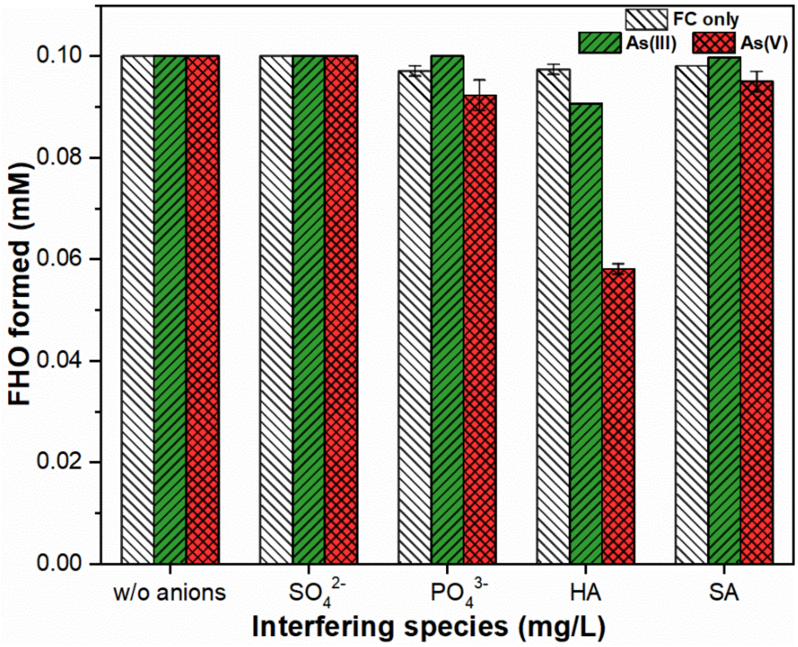

Figure S6. FHO formation under the influence of various interference species.
